# My partner wants a child: A cross-sectional study of the determinants of the desire for children among mutually disclosed sero-discordant couples receiving care in Uganda

**DOI:** 10.1186/1471-2458-10-247

**Published:** 2010-05-13

**Authors:** Jolly Beyeza-Kashesya, Anna Mia Ekstrom, Frank Kaharuza, Florence Mirembe, Stella Neema, Asli Kulane

**Affiliations:** 1 Department of Obstetrics/Gynaecology, Makerere University College of Health Sciences, Kampala, Uganda; 2 Department of Public Health Sciences, Karolinska Institutet, Stockholm, Sweden; 3 Makerere University Institute of Social Research, Kampala, Uganda

## Abstract

**Background:**

The percentages of couples in HIV sero-discordant relationships range from 5 to 31% in the various countries of Africa. Given the importance of procreation and the lack of assisted reproduction to avoid partner transmission, members of these couples are faced with a serious dilemma even after the challenge of disclosing their HIV status to their spouses. Identifying the determinants of the decision to have children among sero-discordant couples will help in setting reproductive intervention priorities in resource-poor countries.

**Methods:**

We conducted a survey among 114 mutually disclosed sero-discordant couples (228 individuals) receiving HIV care at four centres in Greater Kampala, between June and December 2007. The data we collected was classified according to whether the man or the woman was HIV-positive. We carried out multivariate logistic regression modelling to determine factors (age, gender, and the influences of relatives and of health workers, ART knowledge, and disclosure) that are independently associated with a desire for children.

**Results:**

The majority, 59%, of the participants, desired to have children. The belief that their partner wanted children was a major determinant of the desire to have children, irrespective of the HIV sero-status (adjusted odds ratio 24.0 (95% CI 9.15, 105.4)). Among couples in which the woman was HIV-positive, young age and relatives' expectations for children were significantly associated with increased fertility desire, while among couples in which the man was positive; knowledge of ART effectiveness was associated with increased fertility desire. Availability of information on contraception was associated with decreased fertility desire.

**Conclusions:**

The gender of the positive partner affects the factors associated with a desire for children. Interventions targeting sero-discordant couples should explore contraceptive choices, the cultural importance of children, and partner communication.

## Background

As the HIV pandemic matures, 75% of infected people are of reproductive age. In Africa, about 5 to 31% of married or cohabitating couples live in a sero-discordant relationship [1-5]. In Uganda, incidence modelling revealed that 43% of all new HIV infections in adults (15-49 years) in 2008 were among people in discordant monogamous relationships [6]. Millions of cohabiting couples do not know each other's HIV status, while sexual relationships remain a challenge in mutually disclosed sero-discordant couples [7]. The desire to have children, societal expectations, and stigmatization of both HIV and infertility all put pressure on sero-discordant people to practice unsafe sex in order to conceive [8]. Despite this, prevention programmes to protect non-infected partners in sero-discordant couples are not well-established. A large body of knowledge exists on the desires and intentions to have children by HIV-positive people in both high and low-resource settings [9-13]. Fewer HIV-positive people in high-income countries desire parenthood than in low-resource countries [12-15]. African men and women living in France were more likely to desire children than their European counterparts, showing how important reproduction is among African populations [13]. Improvements in assisted reproduction technologies, the availability of various fertility options for couples living with HIV, and the effective prevention of mother-to-child transmission have made it more likely that HIV-infected people will choose to have children [10,16]. Low-cost technologies such as teaching people about ovulation and timed intercourse, and self-insemination for positive women with negative partners are readily available [17]. Furthermore, improved access to ART (avoiding giving teratogenic drugs to women of fertile age) provides sero-discordant couples with further options, since the reduction of viral load significantly reduces the risk of sexual HIV transmission [17-20]. Ironically, the knowledge and availability of various reproductive options is lowest in settings with the highest proportion of HIV-discordant couples, where procreation plays the greatest role, and where assisted reproduction is not affordable [14,15]. In African countries with weak health systems, such as Uganda, only one third of HIV-positive pregnant women have access to prevention of mother-to-child transmission of HIV (PMTCT) services [21]. In addition, only 40% of pregnant women have access to skilled attendance at birth [22]. Similarly, most people cannot afford formula feeding, and there is stigma associated with not breastfeeding among those who can afford it [23,24].

The process of rolling out ART has made it possible for millions of HIV-infected people to live longer and healthier lives, and many will contemplate childbearing, but only half of those in sub-Saharan Africa who need ART have access to it. However, sero-discordant couples face the dilemma of possible horizontal and vertical HIV transmission. There is hardly any literature that describes the desire for children among mutually disclosed sero-discordant couples. This paper describes the determinants of fertility decision-making among mutually disclosed sero-discordant couples who have access to ART and PMTCT services in Uganda.

## Methods

### Study settings and sites

The Kampala and Wakiso districts are located in central Uganda. The Kampala district is also the capital city of Uganda, and its estimated population is 1.2 million. Kampala City has multi-cultural, multi-ethnic and diverse socio-economic groupings, and is largely urban and peri-urban. Wakiso has a population of about one million, and the population is predominantly of one ethnic group (77% Baganda) [25]. It surrounds the Kampala district and is more rural than urban. The Kampala and Wakiso districts were selected from the country's 76 districts, since these districts include large urban, peri-urban and rural populations and they have high HIV/AIDS prevalences (3% among 15-24 year olds and 6-10% among those 25+ years) [3]. We recruited sero-discordant couples from four major HIV/AIDS care centres serving the Kampala and Wakiso districts, namely: The AIDS Support Organization (TASO) - Mulago, Infectious Disease Clinic - Mulago Hospital (ISS clinic), Joint Clinical Research Centre (JCRC), and AIDS Information Centre (AIC).

TASO, founded in 1987, has 11 centres throughout Uganda. It provides services, including counselling services, for individuals and families infected or affected by HIV/AIDS, and it provides medical services that focus on treating opportunistic infections and providing ART. It also aims to increase the capacity of staff, grass-root communities and districts for basic HIV/AIDS management, and provides AIDS education and advocacy for people living with HIV/AIDS. In addition, the organisation provides condoms with the dual goals of preventing sexually transmitted diseases and preventing unwanted pregnancies. A few clients receive hormonal contraception. Those who need surgical contraception are referred to specialized units and pregnant mothers are referred for PMTCT [26]. The ISS clinic is an HIV/AIDS clinic at the Mulago national referral hospital, and operates on the same principles in the provision of care. JCRC is a centre of excellence for AIDS care, treatment, research, and training that was founded at the height of the AIDS crisis in Uganda in 1990 to provide HIV/AIDS care for the Ugandan armed forces [27]. AIC started in February 1990 in response to a growing demand for HIV testing. AIC Kampala is one of eight regional branches in Uganda [28]. By 2003, over 5,000 discordant couples had sought voluntary counselling and testing at AIC, although only 150 discordant couples were attending the HIV-discordant people's club at AIC by 2007. All of these units have recently noted that cases of HIV discordance among couples are increasing, and have started "discordant couple clubs" to support couples socially and psychologically. In addition, they provide education on treatment options and on how to prevent the infection of the HIV-negative partner, and they provide an environment for peer psychosocial support groups through the sharing of experiences and the promotion of positive living. These HIV/AIDS care units schedule visits for their clients to attend for follow up and for other activities, even when the clients are well.

### Sampling and recruitment of study participants

In June 2007, 348 sero-discordant couples were registered with TASO, JCRC, the ISS clinic and AIC (113, 50, 35, and 150 couples, respectively). We included only couples in which at least one partner was aged 40 years or younger because most Ugandan women will by then have completed childbearing [22]. Other eligibility criteria included that the person had known sero-status for more than six months and that the person should not be involved in any behavioural studies that could affect fertility decisions. We identified 150 eligible couples. We used the method developed by Kish and Leslie for descriptive cross-sectional studies. The sample size required, n, is given by n = pqz^2^/d^2^, where p is the proportion the desire for children among HIV discordant couples in Uganda is 0.5, q = 1-p = = 0.5, z is 1.96 (for 5% alpha error) and d is precision which is 0.05.

The value of p, the desire for children among HIV discordant couples in Uganda, is unknown, so we guessed that it is 50%. This gives a requirement for 384 couples, if the population available for sampling is infinite. However, the eligible population is finite, 150 couples. Therefore, we used the modified formula that is used for a finite population: n/(1+ (n-1)/N)), where N is the population size (150), and n = 384. We guessed the non-response rate would be 5%. The sample size required now became 114 couples.

From June to December 2007, we consecutively recruited and obtained informed consent from 114 sero-discordant couples receiving care at the four HIV care units. Our index client was a positive person who was asked to bring his/her partner at an appointed time, or give us his/her telephone number to enable us to contact them. We approached 134 index clients (HIV-infected) for interview; fifteen clients were dropped out of analysis because their HIV-negative spouses refused to participate in the study, while 5 HIV-infected clients declined to participate in the study. Thus, we recruited 114 index HIV-positive participants and their 114 negative partners. The distribution among the genders was nearly equal: 59 men and 55 women were HIV-infected. Each participants was interviewed separately, and analysed as an individual within the couples' gender-HIV status group.

Data were collected using a structured questionnaire that contained both open and closed-ended questions. Factors that may influence the outcome of fertility decision-making were recorded. These factors included: socio-demographic characteristics, the belief that their partner desired children, discussion with spouse about pregnancy, number of children desired, the use of ART, and knowledge about PMTCT. Further factors that were recorded were the role of stigma, influence of significant others (family, friends and health workers), the effect of disclosure to relatives, whether they had had discussions with health workers about pregnancy, contraception and condom use, and whether it was the man or the woman who was HIV-positive. Category responses for the personal or partner's believed desire to have children were recorded as: "Not at all", "I don't know", "Maybe I/he/she wants", and "Definitely I/he/she wants". Questions on occupation and type of contraception used were open-ended. Participants were asked what contraceptives they were using. Each reply was followed by a "What else?" question, in order to gain information about dual methods of contraception.

### Ethical considerations

The study was approved by the Faculty of Medicine Research and Ethics Committee, and Uganda National Council of Science and Technology (NCST). Informed consent was obtained from all participants before the interview.

### Data analysis

All data were entered using Epi Info version 3.4. Analysis was done using STATA version 10. We defined the desire to have children as the study outcome. The response rate for "I don't know" was very low, 5/228 (2.2%), and this reply was interpreted as no desire for children and combined with "Not at all". The responses "Maybe I/he/she wants" and "Definitely I/he/she wants" expressed the desire to have children. The question concerning condom use during the last 12 months had options of "Always used a condom", "Sometimes used a condom", and "Never used a condom". The responses "Sometimes" and "Never" were combined to represent "inconsistent use" and the reply "Always" to represent "consistent use". Open-ended responses on occupation and type of contraception were coded into categories before they were included in the quantitative analysis. Analysis of the replies to other open-ended questions is not presented in this manuscript. We grouped the data according to the gender of the HIV-infected individual in the couple (59 HIV-infected men with their negative wives in one group, and 55 HIV-infected women with their negative husbands in the other). Positive-woman couples were taken to be the reference group. We carried out non-conditional univariate and bivariate logistic regression analyses to calculate odds ratios (OR) and their respective 95% confidence intervals (95% CI), in order to identify factors associated with the desire to have children. All variables with *p*-value lower than 0.20 were included in the model. Backward stepwise elimination was carried out while assessing the impact of each variable on the model using the -2 log likelihood ratio test. This procedure assessed the effect of confounding. The model was constructed with only those variables that were significant and those that were potentially plausible. The main predictor of the desire to have children ("partner wants children") was adjusted for age of the participant, knowledge that ART is effective in reducing MTCT, relatives' desire that the participant produces children, and participants' discussion with health workers about contraception. All *p*-values were two-tailed at a significance level of 5%. Only variables significantly associated with the outcome were retained in the final multivariate models. We also conducted a goodness-of-fit calculation, and tested the model using the Hosmer-Lemeshow chi squared statistic.

## Results

The median age of participants was 33 years (inter-quartile range 28-40 years). Females were younger, 30 years old (inter-quartile range 27-35 years), while males were 38 years (inter-quartile range 30-43 years). Slightly more men (59) than women (55) were the positive partner. Males reported a lifetime number of sexual partners that was much higher, eight (inter-quartile range, 5-15 partners), than that reported by females, three (inter-quartile range 2-5 partners). Most participants (86%) described their current relationship as monogamous, while 14% of the participants acknowledged having two or more partners at the time of the survey. The median duration of the relationship with the primary partner was 7 years. Most participants were of low socio-economic status, with 32% of the men being peasant farmers and 65% of the women being housewives. A total of 41% (52% of men and 31% of women) obtained their income from temporary jobs or casual labour, such as motorcycle transport services, or from small businesses, such as market vending. Only 10% (16% of men and 4% of women) were in salaried professional employment. The majority, (83%), had secondary school education or less. The majority, (93%), of participants had children, (89% of couples in which the woman was the HIV-positive partner, (positive-woman couples); and 97% of couples in which the man was the positive partner, (positive-man couples)). The median number of living children was 3 (inter-quartile range 1-5 children). More positive-man couples had four or more children, (47.5%), than was the case for positive-woman couples, (32.7%), *p *value = 0.024. Slightly more positive-man couples than positive-woman couples had had a child after discovering their sero-status, though the difference was not statistically significant (Table 1).

**Table 1:** The characteristics and other variables of sero-discordant couples, and the gender of the positive partner.

Characteristics	Female + ** N = 110	Male + N = 118	OR* (95% CI)	*p *value
Age of participants				
31-45+ years	59 (53.6)	78 (66.1)		
15-30 years	51 (46.4)	40 (33.9)	0.59 (0.34, 1.02)	0.055
Number of living children				
4-22 children	36 (32.7)	56 (47.5)		
0-3 children	74 (67.3)	62 (52.5)	0.54 (0.31,0.93)	0.024
Had children after knowing status				
Yes	29 (26.4)	39 (33.0)	1.38 (0.78, 2.44)	0.271
No	81 (73.6)	79 (67.0)		
Participant desires to have children				
Yes	70 (63.6)	65 (55.1)	0.70 (0.41, 1.19)	0.190
No	40 (36.4)	53 (44.9)		
Belief that the partner wants to have children				
Yes	74 (67.3)	67 (56.8)	0.64 (0.37, 1.10)	0.104
No	36 (32.7)	51 (43.2)		
Combined couple's desire to have children				
Yes	81 (73.6)	76 (64.4)	0.65 (0.37, 1.14)	0.134
No	29 (26.4)	42 (35.6)		
Number of further children planned***				
1	18 (26.1)	30 (48.4)	2.66 (1.28, 5.53)	0.009
2 or more	51 (73.9)	32 (51.6)		
Relatives want you to get children				
Yes	62 (56.4)	64 (54.2)	0.92 (0.54, 1.55)	0.747
No	48 43.6)	54 (45.8)		
Disclosed to relatives				
Yes	64 (58.2)	86 (72.9)	1.93 (1.11, 3.37)	0.020
No	46 (41.8)	32 (27.1)		
Are you using contraception?				
Yes	69 (66.4)	88 (75.2)	1.54 (0.86, 2.76)	0.148
No	35 (33.6)	29 (24.8)		
Type of contraceptive used				
Condoms	61 (85,9)	84 (92.3)	2.41 (0.68, 8.60)	0.175
Condom + other	3 (4.2)	3 (3.3)	1.75 (0.23, 13.2)	0.587
Other methods (pills, injections, BTL, etc.)	7 (9.9)	4 (4.4)		
Condom use				
Consistent use (always)	55 (50.0)	72 (61.0)		
Inconsistent use (sometimes/not at all)	55 (50.0)	46 (39.0)	1.57 (0.93, 2.65)	0.095
Self or partner on ART				
Yes	43 (39.1)	56 (47.5)	1.41 (0.83, 2.39)	0.204
No	67 60.9)	62 (52.5)		
How effective is ART? (More than 70%)				
Yes	45 (45.9)	48 (48.0)	1.09 (0.62. 1.90)	0.770
No	53 (54.1)	52 (52.0)		
Number of care episodes in the last six months				
More than three times	42 (38.2)	39 (33.1)		
Twice or Less	68 (61.8)	79 (66.9)	1.25 (0.73, 2.16)	0.419

Note:

* Continuous variables have been dichotomised to calculate odds ratios

** Female-positive couples were the reference group.

*** Includes only those who planned to have children, therefore missing values for those who did not plan to have children.

Only 43% of HIV-infected individuals were taking ART. The median duration of taking ART was 2 years. Most participants were in good physical health. In reply to a question how often they had sought health care in the preceding six months, 64.5% reported seeking care on two or fewer occasions. Participants were more likely to want to have children if they had not been ill or if they had been ill and sought care less than twice in the preceding six months, OR 2.80 (95% CI 1.57, 4.99), *p *value = 0.0003. Most (88%) of the participants knew about the existence of HIV drugs for PMTCT and ART, but only 47% believed that ART/PMTCT was more than 70% effective in preventing vertical transmission of HIV. However, significantly fewer positive-man couples (64%) than positive-woman couples (81%) reported that this knowledge had played a role when making a fertility decision, *p *= 0.008 (Table 1).

### Fertility decisions

Most participants (59%) desired to have children sometime in the future, with 43.4% replying that they "Definitely want" children, and 15.8% replying that they "Might want" children. "Not at all" was the response of 40.8%. Some participants, 12%, were pregnant or their partners were pregnant at the time of interview, although three of these had not planned to get pregnant. Positive-woman couples (64%) were more likely to desire a child than positive-man couples (55%), but the difference was not statistically significant (*p *= 0.190). A follow-on question asked about the number of children the participant planned to have, and 97% of participants reported that they planned to have a definite number of children. The median number of more children planned was two. Positive-woman couples were more likely to plan to have more children (73.9%) (67.7% of positive women and 78.9% of negative men) than positive-man couples (51.6%) (positive men, (54.8%) and negative women, (48.4%)), *p *= 0.009. Most participants (71%) replied that they were not in a hurry to get pregnant, while 29% were currently trying to become pregnant. The average planned delay for the 71% who planned a pregnancy later was 1.6 years (SD 1.8 years). The most common type of contraceptive used was the condom (90%): less than 4% used dual methods. Slightly over half (56%) of the participants reported using condoms consistently, 30% used condoms sometimes and 14% never used condoms.

Sixty-seven percent of participants in positive-woman couples believed that their partner wanted children, while this was the case for only 57% of participants in positive-man couples. Only five participants answered "I don't know" to the question of whether the partner wanted children, and these were included in the group that had no desire for children. These were three positive males, one negative female and one positive female. Since we had the replies from both members of a couple, we were able to compare the partner's belief in a participant's fertility desire and the participant's actual desire. This belief was more than 80% correct. In all, 64% reported that knowing that their partner wanted children influenced them in their desire to have children. Overall, 36% had discussed with their partner when to get pregnant, and having held such a discussion was significantly associated with the desire to have children only in the group of couples in which the woman was positive, *p *= 0.003.

Even though 55% of participants reported that their relatives wanted them to have children, 82% thought that this would not be the case if the relatives knew that one member of the couple was HIV-positive. However, 69% of participants believed that their relatives would still care for them during childbirth even if they knew they were HIV-positive. More positive-man couples, 73%, (84.6% of positive men and 61.0% of negative women) had revealed their HIV status to at least one relative than was the case for positive-woman couples (58%) (81.8% of positive women and 54.6% of negative men), *p *= 0.02, Table 1. In addition, 90% of the participants believed they would obtain support from health workers/counsellors if they became pregnant.

Positive-man couples and positive-woman couples were analysed separately (Tables 2 and 3) in order to assess the effect of gender and HIV status on fertility decisions. Significant factors that influenced the decision whether to have children in both groups were: younger age (less than 30 years), having three or fewer living children, the belief that their partner wanted children, and pressure from relatives for the couple to have a baby. Further factors that were important in positive-woman couples were not having disclosed HIV status to relatives, and having held discussions with the partner on when to get pregnant. Further factors that were important in positive-man couples were possessing the knowledge that ART/PMTCT is more than 70% effective and having held discussions with health workers about contraception.

**Table 2:** Bivariate and multivariate regression analysis of factors associated with the desire to have children in couples in which the woman is the positive partner.

Factors	Desire for children	Crude OR (95% CI)	*p *value	Adjusted OR (95% CI)	*p *value
	**N (row %)**				

Age of participant					
31-45+ years	32 (54.2)	1.0			
15-30 years	38 (74.5)	2.45(1.07, 5.68)	0.028	3.33 (1.03, 10.8)	0.045
Number of living children					
4-22 children	14 (38.9)	1.0			
0-3 children	56 (75.7)	4.89 (1.95, 12.3)	0.000	-	-
Belief that the partner wants to have children					
No	7 (19.4)	1.0			
Yes	63 (85.1)	23.7 (6.05, 93.1)	0.000	26.3 (7.89, 87.6)	0.000
Self or partner on ART					
No	23 (53.5)	1.0			
Yes	47 (70.2)	2.04 (0.91, 4.60)	0.078	-	-
How effective is ART? (More than 70%)					
No	33 (62.3)	1.0			
Yes	30 (66.7)	1.21 (0.52, 2.80)	0.652	-	-
Relatives want you to get children					
Yes	20 (41.7)	1.0			
No	50 (80.7)	5.83 (2.29, 14.9)	0.000	6.77(1.12, 21.7)	0.001
Wants their HIV sero-status to remain a secret					
No	18 (50.0)	1.0			
Yes	52 (70.3)	2.36 (1.02, 5.49)	0.039	-	-
Disclosed to relatives					
No	35 (76.1)	1.0			
Yes	35 (54.7)	0.38 (0.16, 0.90)	0.022	-	-
Discussed when to get pregnant					
No	36 (52.9)	1.0			
Yes	34 (80.9)	3.78 (1.46, 9.75)	0.003	-	-
Discussed with Health workers about:					
Contraception and HIV					
No	25 (65.8)	1.0			
Yes	45 (63.4)	0.90 (0.39, 2.06)	0.803	-	-
Pregnancy and HIV					
No	29 (61.7)	1.0			
Yes	41 (66.1)	0.62 (0.28, 1.39)	0.242	-	-

Note: OR: odds Ratio. CI: confidence interval

Predictive capability = 83.6%, and the Hosmer-Lemeshow goodness-of-fit test between predicted and observed probabilities is *p *= 0.91, which is not significant.

**Table 3:** Multivariate logistic regression analysis for factors associated with the desire to have children in couples in which the man is the positive partner.

Factors	Desire to have children	Crude OR (95% CI)	*p *value	Adjusted OR (95% CI)	*p *value
	**N (row %)**				

Age of participant					
31-45+ years	37 (47.4)	1.0			
15-30 years	28 (70.0)	2.59(1.13, 5.94)	0.020	-	-
Number of living children					
4-22 children	19 (33.9)	1.0			
0-3 children	46 (74.2)	5.60 (2.34, 13.4)	0.000	-	-
Belief that the partner wants to have children					
No	9 (17.6)	1.0			
Yes	56 (83.6)	23.8 (6.55, 86.2)	0.000	24.0 (9.15, 105.4)	0.000
Self or partner on ART					
No	29 (55.8)	1.0			
Yes	36 (58.1)	1.29 (0.62, 2.68)	0.495	-	-
How effective is ART? (More than 70%)					
No	24 (46.2)				
Yes	33 (68.8)	2.57 (1.10, 5.97)	0.023	3.66 (1.15, 11.7)	0.029
Relatives want you to get children					
Yes	21 (38.9)	1.0			
No	44 (68.8)	3.46 (1.55, 7.70)	0.001	-	-
Wants their HIV sero-status to remain a secret					
No	17 (47.2)	1.0			
Yes	48 (58.5)	1.58 (0.71, 3.50)	0.257	-	-
Disclosed to relatives					
No	20 (62.5)	1.0			
Yes	45 (52.3)	0.66 (0.28, 1.52)	0.325	-	-
Discussed when to get pregnant					
No	41 (52.6)	1.0			
Yes	24 (60.0)	1.35 (0.62, 2.95)	0.444	-	-
Discussed with Health workers about:					
Contraception and HIV					
No	30 (66.7)	1.0			
Yes	35 (47.9)	0.46 (0.21, 1.01)	0.048	0.29 (0.08, 0.96)	0.042
Pregnancy and HIV					
No	27 (60.0)	1.0			
Yes	38 (52.1)	0.72 (0.34, 1.54)	0.401	-	-

Note: OR: odds Ratio. CI: confidence interval.

Predictive capability = 83.0%; and the Hosmer-Lemeshow goodness-of-fit test between predicted and observed probabilities is *p *= 0.06 which is not significant.

Factors that remained significant in positive-man couples in the multivariate analysis were: the belief that the partner wants children, adjusted OR 24.0 (95% CI 9.2, 105.4), having held discussions with health workers on contraception, adjusted OR 0.3 (95% CI 0.1, 1.0), and knowledge of the high efficacy of ART, adjusted OR 3.7 (95% CI1.2, 11.7) (Table 2). Factors that remained significant in positive-woman couples were: the belief that the partner wants children, adjusted OR 26.3 (95% CI 7.9, 87.6), pressure from relatives to have children, adjusted OR 6.8 (95% CI 1.1, 21.7), and age of 30 years or less, adjusted OR 3.3 (95% CI 1.0, 10.8) (Table 3).

## Discussion

This study has shown that many sero-discordant couples in Uganda desire to have children. The major determining factor in the decision-making process, irrespective of the gender of the positive person within the couple, is the belief that the partner wants to have a child. Other factors that influence the pregnancy decisions of sero-discordant couples differ between positive-woman couples and positive-man couples. Sero-discordant couples are a special at-risk group for HIV transmission and acquisition. A strong desire for reproduction may reduce the risk of partner infection to a secondary concern [8]. Few studies have examined the context of reproductive decision-making and the desire for parenthood among sero-discordant couples [12,13]. Our results of the desire for children is comparable to that in other studies among HIV infected people who still have partners and those in the era of ART scale-up [10,14,29].

Our study has shown that couples are strongly influenced by their partners and a member of a couple is more likely to desire children if he or she believes that the partner wants children. This agrees with previous studies [9,15,30]. We compared the belief whether a partner wanted children with the actual desire since we had data from both partners. The belief was correct in more than 80% of cases. Only 36% of the couples had discussed pregnancy issues, and we conclude that there is a high level of non-verbal communication in couples with HIV. This supports the framework of modelling fertility motivation proposed by Miller at al. [31], which shows that a partner in a couple is able to perceive their partner's desires through non-verbal cues. However, a partner's fertility desire may be overestimated [32]. Nonetheless, health workers can exploit and strengthen this non-verbal communication to assist sero-discordant couples in making informed reproductive decisions. Male (partner) involvement is critical for the success of HIV/AIDS prevention and for the implementation of reproductive health programmes [33]. More emphasis must, therefore, be placed on helping men understand their strategic position in HIV-prevention efforts both using active community information and using targeted information to sero-discordant couples.

Pressure from relatives is a major cause of the desire to have children among positive-woman couples. Relatives are often eager to see couples have children and are part of the decision-making process [34]. They frequently lack, however, knowledge of the person's HIV status [7]. Participants in this study believed that relatives might not want them to get pregnant if they knew that at least one of the couple was HIV-infected. Those who did not disclose their HIV status were more likely to desire a child, especially among the positive-woman couples. Perceived or experienced stigma leads HIV-infected people to choose to get pregnant and live a normal life, in order to avoid the community stigma associated with the suspicion of HIV-infection [35]. A very high proportion of HIV-positive men (85%) disclosed their HIV status to relatives. We have not collected data concerning the reasons for disclosure. It is possible that a man will disclose his HIV status in order to gain social support, or to inform relatives in preparation for the care of his family when he dies.

A belief in the high efficacies of ART and PMTCT is a strong motivator for childbearing in positive-man couples. ART improves health and rekindles hope for a longer life. This may lead HIV-positive men to consider having more children since they now have time to raise the children. We have previously shown that an important consideration for men is to secure lineage and posterity [8]. Recent studies have shown that the desire for children increases when people take ART [29,35,36]. In addition, people on ART choose to use methods of contraception of a less permanent nature [11,35]. Ninety percent of our participants used condoms as the only contraceptive method, although the effect of using ART on contraception was not significant (data not shown). We have, however, previously shown that couples who desire to have children use condoms inconsistently [8]. ART reduces the risk of HIV transmission through a reduction in viral load [17-20]. Nonetheless, some studies have shown that high-risk sexual behaviour increases in people on ART, thus increasing the risk of transmission [37]. Even so, health workers should explain the window of opportunity that ART gives to those who want to conceive. They should emphasise the fact that ART does not completely eliminate the risk of transmission and that it is critical to review ART (if the woman is positive) in order to minimise its potential effects on the mother and unborn baby[3].

It is interesting that discussions with health workers about childbearing did not significantly influence pregnancy decision-making. A previous qualitative study with a subgroup of discordant couples reported that health workers had not given sufficient advice about childbearing [8]. Other studies have shown that health professionals' advice does not affect the decision to have children because the participants believe that health professionals will be negative towards childbearing [30,38,39]. We need to understand what it is about childbearing discussions that causes resentment, because participants in the qualitative study mention health workers as a source of guidance [8]. If patients shun the advice of health workers about childbearing, the full potential benefits of PMTCT and ART will not be realised. It should be emphasized that the sexual and reproductive rights of people living with HIV/AIDS (PLWHA) cannot be negotiated. However, several studies have shown that health workers are ambivalent about the reproductive rights of HIV-positive persons [38-40]. One study in Vietnam, however, showed that health workers there support childbearing for PLWHA because they understand the cultural context of childbearing and lineage continuation [41]. Shortages of health workers in low resource countries, low or unpaid salaries, and poor training, supervision, and working conditions were mentioned as factors that hinder health workers from helping people with HIV/AIDS to claim their sexual and reproductive health rights [42]. Further obstacles are policies that are difficult to understand and non-prescriptive guidelines: these leave PLWHA confused about how to get help from health professionals.

This was a cross-sectional study, which means that causal and temporal relationships are difficult to establish. Decision-making is a process, and decisions made may change over time. Uganda's sero-behavioural survey [3] showed that HIV is more prevalent in the wealthiest quartile of people, while the majority of our study group were poor. Couples with higher incomes may be attending private clinics and thus be under-represented in our study. We selected study participants who had attended several seminars on discordance because we believed that they would be able to freely talk about their experiences and plans. This might not be the case for couples who do not frequent the health services for counselling and support. In addition, the experiences and decisions of this study group may not reflect the experiences and decisions of all sero-discordant couples regarding childbearing, but they do reflect those of other sero-discordant couples of a similar age and in similar socio-cultural situations and settings. However, the socio-demographic characteristics, such as median age, years of cohabitation and number of living children, of the study population are similar to those of other studies among discordant couples in sub-Saharan Africa that include Uganda, and we therefore believe that our findings can be generalised to sero-discordant couples in stable relationships [3,7,43].

## Conclusions

This study shows that many sero-discordant couples in Uganda desire to have children. The group of mutually disclosed couples is rarely studied, but it is increasingly important for future interventions that aim to reduce HIV transmission. Openness concerning reproductive issues should be encouraged and health workers should, in a non-directive and non-judgmental manner, assist couples to identify their reproductive options and make informed decisions. The current content of reproductive discussions that occur between health workers and their clients is under-researched. Health systems must be improved, to provide special sexual and reproductive health services tailored to PLWHA, and in particular to people living in HIV discordance. Such services include: dual protection, information concerning the use of ART and hormonal contraceptives and pregnancy, and counselling on preconception strategies. In addition, harm reduction has been proposed as a strategy for discordant couples who want to conceive, but there are few studies on its implementation. Further work must be carried out to determine whether these harm-reduction strategies are effective, before they can be adopted. Policy-makers should also include low-cost assisted reproductive technologies to prevent HIV transmission to the negative partner in HIV care programmes. It is vital to include people living in sero-discordant relationships as a group with special reproductive needs in the National Policy on Sexual and Reproductive Health and Rights, and it is vital to include them in dialogue and when establishing programmes for their needs. Above all, empowering health workers with resources, information, skills and sensitivity-training related to the specific needs of HIV-positive people is critical to securing the sexual and reproductive rights of PLWHA.

## Competing interests

We declare that there are no conflicting interests: financial, political, personal, religious, ideological, academic, intellectual, commercial or otherwise.

## Authors' contributions

JBK and FM conceived the idea, all authors participated in the design of the study, JBK collected the data, supervised by FM and FK. All authors carried out data analysis; and all read, edited and approved the manuscript.

## Pre-publication history

The pre-publication history for this paper can be accessed here:

http://www.biomedcentral.com/1471-2458/10/247/prepub
